# Screening for Tuberculosis Infection among Migrants: A Cost-Effectiveness Analysis in the Italian Context

**DOI:** 10.3390/antibiotics12040631

**Published:** 2023-03-23

**Authors:** Giulia Russo, Valentina Marchese, Beatrice Formenti, Claudia Cimaglia, Gianluca Di Rosario, Irene Cristini, Paola Magro, Issa El-Hamad, Daniela Maria Cirillo, Enrico Girardi, Alberto Matteelli

**Affiliations:** 1 Department of Infectious and Tropical Diseases, University of Brescia and ASST Spedali Civili of Brescia, 25123 Brescia, Italy; 2 Emerging Bacterial Pathogens Unit, IRCCS San Raffaele Scientific Institute, 20132 Milan, Italy; 3 Department of Clinical and Experimental Sciences, WHO Collaborating Centre for Tuberculosis Prevention, University of Brescia, 25123 Brescia, Italy; 4 Department of Infectious Diseases Epidemiology, Bernhard Nocht Institute for Tropical Medicine (BNITM), 20359 Hamburg, Germany; 5 UNESCO Chair “Training and Empowering Human Resources for Health Development in Resource-Limited Countries”, Department of Clinical and Experimental Sciences, University of Brescia, 25123 Brescia, Italy; 6 Clinical Epidemiology Unit, Lazzaro Spallanzani National Institute for Infectious Diseases-IRCCS, 00149 Rome, Italy

**Keywords:** tuberculosis, migrant, Italy, TB infection, cost-effectiveness, screening

## Abstract

Background: Screening of tuberculosis infection (TBI) among migrants from high-incidence countries is a cornerstone of tuberculosis control in low-incidence countries. However, the optimal screening strategy has not been defined yet. Methods: A quasi-experimental study involving migrants residing in the province of Brescia was carried out that aimed at assessing the completion rate, time to completion, preventive treatment initiation rate, and cost-effectiveness of two strategies for TBI screening. They underwent TBI screening with the IGRA-only strategy (arm 1) or with the sequential strategy (tuberculin skin test, TST, followed by IGRA in case of a positive result—arm 2). The two strategies were compared in terms of screening completion, time to complete the screening process, therapy initiation, and cost-effectiveness. Results: Between May 2019 and May 2022, 657 migrants were evaluated, and 599 subjects were included in the study, with 358 assigned to arm 1 and 237 to arm 2. Screening strategy was the only factor associated with screening completion in a multivariable analysis, with the subjects assigned to the IGRA-only strategy more likely to complete the screening cascade (n = 328, 91.6% vs. n = 202, 85.2%, IRR 1.08, 95% CI (1.01–1.14), *p* = 0.019). The time to complete the screening process was significantly longer for patients assigned to the sequential strategy arm (74 days vs. 46 days, *p* = 0.002). Therapy initiation did not significantly differ between the two arms, and cost-effectiveness was higher for the sequential strategy. Conclusion: Sequential strategy implementation for TBI screening among migrants may be justified by its higher cost-effectiveness in spite of the lower completion of the screening cascade.

## 1. Introduction

Recently arrived migrants moving from high-incidence tuberculosis (TB) areas are considered an at-risk group for progression from tuberculosis infection (TBI) to active disease, particularly within the first 5 years of arrival [1].

In Italy, which is among the countries with low incidence of TB (<10/100,000), the TB notification rate was 3.8/100,000 in 2020, with more than half of the cases diagnosed in patients of foreign origin and 71.8% of pulmonary cases [2]. Several studies conducted in Brescia, Northern Italy, confirmed that foreigners represent the group with the highest number of cases also in the study area [3], where TB prevalence and incidence were 545/100,000 persons and 220/100,000 person-years, respectively, in asylum seekers [4]. Noteworthy, Italy has recently shown higher screening yield for active TB among migrants compared with other European countries, ranging from 535/100,000 to 653.6/100,000 in two different screening strategies adopting chest-X-ray [5,6] and 369/100,000 when based on symptom screening and on-spot sputum Xpert Ultra analysis at first arrival [7].

Although there is evidence of the effectiveness of incorporating TBI testing into screening programs for migrants from high TB incidence countries [8,9,10], there is still a vast heterogeneity of implementation practices and lack of consensus on their cost-effectiveness [11,12,13,14,15]. Nevertheless, with regard to the efficacy of TBI treatment in preventing active disease in the migrant population, data from a recent retrospective study published in England on screening to treatment of migrants confirm WHO recommendations, with an 86% reduction in the risk of disease progression in IGRA-positive treated migrants compared with untreated migrants [16,17]. In accordance with the WHO guidelines on the management of tuberculosis infection, the diagnosis of TBI can be made with either the tuberculin skin test (TST) or the interferon gamma release assay (IGRA) test [17,18]. The two tests have a similar positive predictive value for the development of future disease [17,18]. Similarly, the ECDC recommends offering TBI screening using TST or IGRAs immediately after arrival for all migrant populations from countries with a high incidence of tuberculosis [11].

In Italy, the offer of the TST or, alternatively, an IGRA test is recommended (the latter indicated in particular in cases of previous vaccination) for migrants from highly endemic countries [19]. Additionally, in the Lombardy region of Italy, a protocol [20] is applied that recommends sequential screening with TST that, if positive, is followed by a confirmatory IGRA test in all asymptomatic migrants hosted in reception centers with an intention to stay for at least 6 months. 

The current literature on the cost-effectiveness of testing strategies varies according to population characteristics (age, background, and history of previous vaccination with Bacille Calmette–Guérin, BCG). Some studies reported improved cost-effectiveness of IGRA compared with TST, while others found a greater benefit in the sequential strategy (TST + IGRA) [21]. However, a recent systematic review highlighted that only 54% of migrants with a positive TBI test completed preventive treatment, raising questions about how successful these programs are in engaging migrants in the screening and treatment pathway [22]. Improving the effectiveness and cost-effectiveness of TBI screening is essential to make this important intervention accepted in political agendas.

The aim of our study is to compare the screening completion rate in the migrant population between two different strategies: the IGRA-only strategy and sequential TST/IGRA one. We also compared the two strategies in terms of participants who completed the screening cascade, participants who started the preventive treatment, time needed to complete the screening cascade, and cost-effectiveness. 

## 2. Materials and Methods

### 2.1. Study Site and Population

This is a quasi-experimental study including adult migrant people residing in the province of Brescia, Northern Italy. Our study population included asylum seekers and undocumented migrants who had arrived in Italy for five years or less. From May 2019 until May 2022, they were screened for TBI at the Infectious Diseases Unit of the ASST Spedali Civili of Brescia. Following National Guidelines, participants were referred for TBI screening by social workers and educators working at the reception centers for asylum seekers in the province of Brescia and by the Unit of Transcultural Medicine and Sexually Transmitted Diseases (Spedali Civili Hospital, Brescia) (UTMSTD). Exclusion criteria included being underage, having a current diagnosis of active tuberculosis, and/or a history of antituberculosis treatment.

### 2.2. Study Design

This prospective quasi-experimental study aimed at assessing the completion rate, time to completion, preventive treatment initiation rate, and cost-effectiveness of strategies for TBI screening among migrants in Italy. The study was designed as a randomized clinical trial, in which participants were randomly assigned to one of two screening strategies: IGRA-only testing strategy (arm 1) or TST followed by IGRA (sequential screening strategy, arm 2). However, during the study implementation, a nationwide shortage of TST product occurred due to the COVID-19 pandemic, and subjects were assigned to arm 2 based on tuberculin availability.

### 2.3. Study Procedures

At the initial evaluation, signs and symptoms for active tuberculosis were investigated, and chest X-ray and microbiological examinations (direct, GeneXpert, and culture on sputum) were prescribed if needed as screening for active disease. Signs and symptoms for extrapulmonary TB were also ruled out. After exclusions of active disease and potential contagiousness, screening for TBI was performed.

For each participant, demographic (sex, age, country of origin, and date of arrival in Italy) and epidemiological (history of past TB or TB contacts) data as well as clinical history were collected. 

#### 2.3.1. Arm 1 Procedures

For participants assigned to arm 1 (visit 1, V1), IGRA testing was carried out using a Quantiferon-TB Gold Plus kit (Qiagen) on venous blood samples, with the analysis performed according to the manufacturer protocol. Results were communicated to the reception centers. In case of a positive result, participants underwent chest X-ray (VR) and a second clinical evaluation to assess the possible contraindications for preventive treatment (VE) and, if necessary, to initiate preventive treatment (VT).

#### 2.3.2. Arm 2 Procedures

Participants assigned to arm 2 underwent intradermal injection of 0.1 mL of purified protein derivative (PPD) containing 5 tuberculin units on the volar surface of the forearm (V1). The area of induration was evaluated after 48–72 h by a trained healthcare worker (V2), and the test was considered as positive when the diameter of the area of induration was at least 10 mm, according to current guidelines [17]. 

Participants with a positive TST underwent IGRA evaluation on venous blood samples (V3) and, in case of a positive IGRA result, a chest X-ray (VR). Results were communicated as soon as available (V4), and, in case of a positive IGRA test with negative chest X-ray, subjects were evaluated for preventive treatment (VE). 

Regardless of the arm, each patient with TBI underwent at least one outpatient consultation (as per the National Health System indications) to begin preventive treatment (VE), in which the following blood tests were prescribed: complete blood count; alanine aminotransferase; aspartate aminotransferase; bilirubin; and HIV, HBV, and HCV serology. A second outpatient consultation (VT) was performed to restitute blood test results and to begin preventive treatment, if appropriate. 

#### 2.3.3. Definitions

Screening completion was defined as follows:

- Arm 1 (IGRA only): in case of a negative IGRA result, screening was considered completed when the IGRA result was communicated; for patients with a positive IGRA test, chest X-ray (VR) and outpatient consultation for preventive therapy (VE) were required.
- Arm 2 (sequential strategy): in case of a negative TST result, participants completed the screening process when the result was evaluated (V2); in case of a positive TST result, screening was defined as completed either when the IGRA result was communicated or when participants underwent chest X-ray (VR) and outpatient consultation for preventive therapy (VE) according to a negative or positive IGRA test result, respectively.

The proportion of participants starting preventive treatment was calculated for each arm as the ratio between the number of participants who were provided with treatment (VT) and the number of participants who were enrolled for TBI screening.

### 2.4. Statistical Analysis 

Descriptive analysis was conducted using medians and interquartile ranges (IQRs) for continuous variables, and counts and percentages for categorical variables. The comparisons between groups were performed using the Mann–Whitney nonparametric test for continuous variables or the chi-square (χ2) test for categorical variables. We classified countries at high TB incidence according to WHO estimates for 2021 (threshold 150/100,000 population) [13]. Univariable and multivariable Poisson regression analyses, with robust variance, were used to identify factors associated with the completion of the screening cascade for tuberculosis infection, calculating incidence rate ratios (IRRs) and their 95% confidence intervals (95% CIs). Univariable and multivariable Poisson regression analyses with robust variance were also used to identify factors associated with the starting of preventive treatment, reporting incidence rate ratios (IRRs) and relative 95% CIs. Multivariable models were adjusted for sex, age at first test (by 5-year increase), arm, and TB incidence in the country of origin. A *p*-value less than 0.05 indicated conventional statistical significance. All statistical analyses were performed using STATA (StataCorp. 2021. Stata Statistical Software: Release 17. College Station, TX, USA: StataCorp LLC).

### 2.5. Cost-Effectiveness Analysis

Costs were considered from the perspective of the National Health System, and only direct individual medical costs were included. We considered the costs of TST, IGRA, clinical evaluation (for visits VE and VT), chest X-ray, and preliminary blood tests for preventive treatment, according to the healthcare tariff list provided by the Lombardy region. In addition to the chest X-ray performed according to the study protocol, also chest X-ray examinations performed for clinical indication (presence of cough lasting more than two weeks, fever, night sweats, and/or weight loss) were considered among the costs. The effectiveness of the two strategies was calculated in terms of the number of people starting preventive treatment, assuming that TBI treatment is a cost-effective intervention *per se*. For each intervention, the average cost-effectiveness ratio (ACER) was calculated as the ratio between the total cost of the intervention and the number of people who started the treatment for each arm. The comparison between the two strategies was conducted until the start of preventive treatment and did not include the societal costs, as well as the costs for treatment provision, follow-up and side effects, and the treatment outcome. 

Assuming a screening completion rate of 80% in the two groups screened with a single test and a possible 10% reduction in the completion rate in the group assigned to sequential screening, with a non-inferiority margin of 20%, an alpha error of 5%, and a power of 80%, 230 people were enrolled in each group.

### 2.6. Ethical Considerations

The study was approved by the provincial ethical committee (protocol number NP 3086) on 26 June, 2018, and each participant signed an informed consent.

## 3. Results

Between May 2019 and May 2022, 657 migrants were evaluated. Among those, 62 were excluded from the study because of age <18 years (2), diagnosis of active TB (10), history of treatment for TB disease (10), or refusal to participate in the study (V1) (40). The final study population included 595 subjects, with 358 assigned to the IGRA-only screening (arm 1) and 237 to the sequential screening strategy (arm 2). The unbalance in the number of people assigned to the two arms can be explained by the shortage of the TST product during the COVID-19 pandemic, preventing, for several months, assignment of persons to arm 2.

Table 1 summarizes the characteristics of the study population. The vast majority of the subjects included in the study were men (91.4%), and more than half of participants were 26 years old or younger. There are no significant differences in terms of age and sex between the two study arms. However, approximately two-thirds of patients assigned to arm 2 arrived in Italy within 2020, while most of the subjects enrolled for arm 1 arrived in Italy after 2020, with a statistically significant difference between the two arms that can be explained by the shortage of TST resources during the COVID-19 pandemic. 

**Figure 1:**
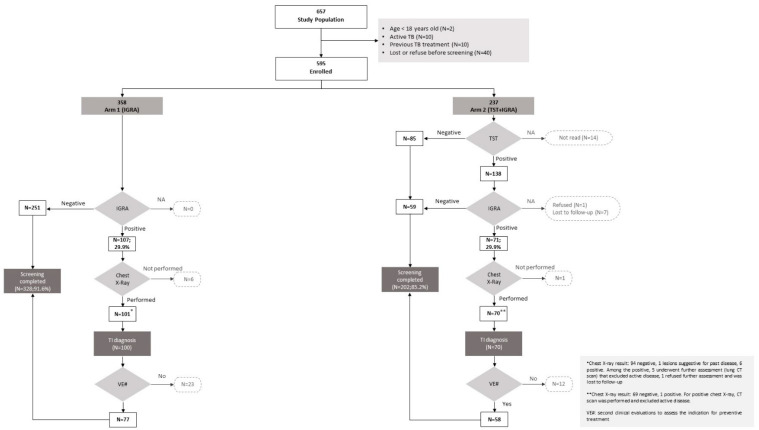
Screening cascade.

In our study population, 530 (89.1%) subjects completed the screening, with 328 (91.6%; n = 328/358) belonging to arm 1 and 202 (85.2%; n = 202/237) assigned to arm 2, as summarized in Figure 1. The rate of positive test results (IGRA for arm 1 and both TST and IGRA for arm 2) was similar in the two groups (29.9%). The rate of completion was statistically significantly higher for arm 1 than for arm 2 (91.6% vs. 85.2%; *p* value = 0.014). Finally, the proportion of eligible subjects who indeed started preventive treatment was not significantly different (18.2%, 65 subjects, for arm 1 and 21.9%, 52 subjects, for arm 2—*p* value 0.256).

The results of the multivariable Poisson regression analysis showed that the subjects enrolled in arm 1 (IGRA-only strategy) were significantly more likely to complete the screening (aIRR = 1.08, 95% CI 1.01–1.14, as compared with those enrolled in arm 2; *p* < 0.019) (Table 2). The screening strategy was the only variable significantly associated with screening completion, while sex, age, and TB incidence in the country of origin, introduced as confounding factors, showed no correlation with the outcome.

For subjects with a positive screening result that completed the evaluation process and reached the evaluation visit (VE), the time (median, days) to complete the screening process was significantly longer in arm 2 than in arm 1 (74 days vs. 46 days, respectively, *p* = 0.002). 

Multivariable Poisson regression analysis was also used to assess the association between preventive treatment initiation and screening strategy, sex, age, and TB incidence in the country of origin. No statistically significant association was found (Table 3).

Finally, the two screening strategies were evaluated in terms of cost-effectiveness, as reported in Table 4. The sequential strategy proved to be the most cost-effective one, being at the same time the most effective and least costly.

## 4. Discussion

Our prospective investigations show that screening migrants for TBI with IGRA alone offers a significant, but operationally small advantage compared with sequential TST + IGRA. Despite the fact that in IGRA-only strategy subjects are more likely to complete the screening, as seen in other settings [24], this strategy is less cost-effective compared with sequential TST + IGRA. 

There are three aspects to consider in assessing the cost-effectiveness of TBI screening among migrants. The first one is whether it is a cost-effective intervention per se. Despite the limited evidence, we assumed the cost-effectiveness of the screening for TBI, in line with the conditional recommendation to consider systematic TBI testing and treatment for immigrants from high-TB-burden countries set out in the WHO guidelines [17,25]. 

The second one is the evaluation of which strategy (IGRA, TST, or sequential TST/IGRA) is the most cost-effective. Sequential screening is the most cost-effective strategy from a healthcare perspective, as well as in comparison with TST or IGRA alone [21]. In the evaluation of cost-effectiveness, test accuracy seems to play a major role [17]: TST accuracy appears to be reduced by BCG vaccination at birth, practiced in many parts of the world [26]. We speculate that the high rate of unconfirmed TST positivity in our sequential arm (42.8% of cases have a negative IGRA test) may be explained by the median young age of our population. 

The third aspect is the evaluation of cost-effectiveness of the different testing strategies among migrants. Similarly to other at-risk groups, evidence is still scarce and considered weak among migrants, claiming for additional studies to address the knowledge gap [15]. In our study, the sequential strategy (TST/IGRA) was more cost-effective than IGRA-only. Our result corroborates evidence from a mathematical model based on a large prospective UK cohort in 2018 [27] in which the sequential strategy resulted to be the most effective strategy for screening among migrants coming from high-incidence countries.

Evidence shows that screening completion and treatment initiation rates influence the cost-effectiveness of screening [28]. In our population, although completion was higher in the IGRA arm, there were no differences in treatment initiation between the two screening procedures. One possible explanation is that patients in the sequential arm require more frequent contacts with healthcare systems since result assessment for TST needs a clinical evaluation. That increases the risk of losses to follow-up but simultaneously can enhance trust in the service and indirectly enhance treatment acceptability. Unfortunately, the absence of qualitative data does not allow a comprehensive evaluation of the phenomenon, which, however, is worthy of further investigation.

The assessment of cost-effectiveness for TBI screening among migrants is affected by the TBI prevalence found in the screened population [29]. In our study, screening was offered to all migrants who recently arrived, asylum seekers or undocumented, regardless of incidence in the country of origin, resulting in around 30% of our population coming from countries with TB incidence <150/100,000. Estimates of TBI prevalence among migrants are heterogeneous both due to the different adopted tests (and their specificity) and the possible risks related to the migration route and type of migration (forced, economic, educational, etc.) [25]. In a recent meta-analysis, prevalence of TBI ranged from 0.4 to 81.5%, with a prevalence higher than 30% in more than 60% of the included studies [30]. The Central Mediterranean is one of the most dangerous migration routes worldwide in which poor, crowded living conditions, sometimes in detention, frequently occur, increasing the risk of new TB infections. In support of that, results from a multicountry screening assessment for active TB among foreign-born people and asylum seekers confirmed a higher screening yield in Italy compared with other European countries [6].

The overall TBI prevalence found (around 30%) is consistent with data of a recent meta-analysis that showed a higher prevalence of TBI among asylum seekers and refugees coming to Europe when compared with the American context (41%, 95% CI, 20–65 vs. 28, 95% CI, 18–40) [30].

Our study has two major limitations. During the study implementation, two major events occurred: the COVID-19 pandemic, with the closing down of most non-urgent outpatient services (UTMSTD included), and the worldwide shortage in tuberculin supply. Those two unpredictable events prevented the systematic randomization of participants. Nevertheless, sample size calculations were respected to assess the cost-effectiveness.

Cost-effectiveness analysis considered costs from the National Health System perspective only, without assessing social and other indirect costs, which could possibly be higher in the sequential arm given the higher number of visits per screened patient (2.48 vs. 1.50 in the IGRA-only arm). The inclusion of those costs in the analysis could have led to different results and to a more comprehensive estimate. However, our population mainly consists of asylum seekers in their first period of stay in Italy, with heterogeneous working and housing conditions, and no individual data were recorded at enrollment. Thus, any analysis including societal and other indirect costs would have been based on approximate data. Despite these limitations, the results of our study can inform Italian policymakers and help to optimize the screening for TBI among migrants, which is currently recommended in the country for people coming from countries with TB incidence >100,000 without any specifications on the testing strategy to be used [19,31,32] resulting in differences within regions and sometimes also provinces.

## 5. Conclusions

Testing for TBI among asylum seekers in reception centers is most cost-effective when adopting a sequential strategy (TST + IGRA). We suggest that this strategy be implemented in the Italian setting on a national scale. Additional studies including the assessment of indirect costs or the evaluation of cost-effectiveness in different reception systems can contribute to a wider generalization of our results.

## Figures and Tables

**Table 1:** Characteristics of study population.

	Arm 1 (IGRA Only)	Arm 2 (TST + IGRA)	Total	*p*-Value
**Total population, n**	358	237	595	-
**Sex, n (%)**				
Men	321 (89.7)	223 (94.1)	544 (91.4)	0.059
Women	31 (10.3)	14 (5.9)	51 (8.6)	
**Median age at the time of arrival, years (IQR)**	26 (22–33)	26 (23–32)	26 (22–32)	0.955
**Age at the time of the first test, n (%)**				
≤26 years	196 (54.8)	127 (53.6)	323 (54.3)	0.781
>26 years	657 (45.3)	110 (46.4)	272 (45.7)	
Year of arrival *				
≤2020	171 (47.8)	160 (67.5)	331 (55.6)	<0.001
>2020	179 (50.0)	46 (19.4)	225 (37.8)	
**TB incidence in the country of origin, n (%) ^**				
<150/100,000	118 (33.0)	90 (38.0)	208 (35.0)	0.209
≥150/100,000	240 (67.0)	147 (62.0)	387 (65.0)	

* Year of arrival was missing for 39 participants, 8 assigned to arm 1 and 31 assigned to arm 2. Percentages were calculated on the population of each arm. ^ Rates per 100,000 population. Global Tuberculosis Report—WHO 2022 [23].

**Table 2:** Incidence rate ratio (IRR) and 95% confidence intervals (CIs) for screening completion.

	Screening Completed/Tot	Univariable		Multivariable	
	(530/595)	IRR (95% CI)	*p*-Value	aIRR (95% CI)	*p*-Value
**SEX**					
Men	485/544	Ref.		Ref.	
Women	45/51	0.99 (0.89–1.10)	0.846	0.97 (0.88–1.09)	0.669
					
					
					
**Age at first test, by 5-year increase**		1.00 (0.99–1.02)	0.657	1.00 (0.99–1.02)	0.712
**ARM**					
1 (IGRA)	328/358	1.07 (1.01–1.14)	0.022	1.08 (1.01–1.14)	0.019
2 (TST + IGRA)	202/237	Ref.		Ref.	
**TB INCIDENCE IN THE COUNTRY OF ORIGIN ^**					
<150/100,0000	185/208	Ref.			
≥150/100,0000	345/387	1.00 (0.94–1.06)	0.939	1.00 (0.94–1.06)	0.969

Abbreviation: IRR, incidence rate ratio; aIRR, adjusted incidence rate ratio; CI, confidence interval; Ref, reference. Model adjusted for all variables included in the table. ^ Rates per 100,000 population. Global Tuberculosis Report—WHO 2022 [23].

**Table 3:** Incidence rate ratio (IRR) and 95% confidence intervals (CIs) for preventive treatment initiation.

	Treatment Initiation/Tot	Univariable		Multivariable	
	(117/595)	IRR (95% CI)	*p*-Value	aIRR (95% CI)	*p*-Value
**SEX**					
Men	111/544	Ref		Ref	
Women	6/51	0.58 (0.27–1.25)	0.161	0.59 (0.29–1.28)	0.181
					
					
					
**Age at the first test, by 5-year increase**		1.01 (0.93–1.11)	0.766	1.03 (0.94–1.14)	0.523
**ARM**					
1 (IGRA)	65/358	0.83 (0.60–1.14)	0.255	0.83 (0.60–1.15)	0.272
2 (TST + IGRA)	52/237	Ref	Ref	Ref	
**TB INCIDENCE IN THE COUNTRY OF ORIGIN ^**					
<150/100,0000	37/208	Ref		Ref	
≥150/100,0000	80/387	1.16 (0.82–1.65)	0.402	1.17 (0.82–1.67)	0.400

Abbreviation: aIRR, adjusted incidence rate ratio; CI, confidence interval; Ref, reference. Model adjusted for all variables included in the table. ^ Rates per 100,000 population. Global Tuberculosis Report—WHO 2022 [23].

**Table 4:** Costs and cost-effectiveness analysis for two screening strategies: IGRA only (arm 1) and sequential screening with TST followed by IGRA (arm 2).

	Cost per Unit	Arm 1 (IGRA)			Arm 2 (TST + IGRA)	
		N.	Costs (€)		N.	Costs (€)
TST *		-			237	1374.60
IGRA		358	18,705.50		130	6792.50
Chest X-ray ^#^		162	2818.80		81	1409.40
VE **(performed as distinct from VT)**		40	900.00		26	585.00
VT **(performed as distinct from VE)**		28	630.00		20	450.00
VE + VT **(performed at a single time point)**		37	832.50		32	720.00
Blood tests		65	2905.50		52	2324.40
Total			**26,792.30**			**13,655.90**
						
Costs per subject undergoing TBI screening			74.84			57.62
Costs for 100 subjects undergoing TBI screening			7483.88			5761.98
ACER ^§^			412.19			262.61

* Costs for TST included both costs for intradermal injection and evaluation after 48–72 h. # Chest X-ray performed for suspect active TB (in addition to those performed as part of TB infection screening): 61 for arm 1 and 11 for arm 2. § ACER (average cost-effectiveness ratio) was calculated as the ratio between the costs and the number of subjects who started treatment for each arm (65 for arm 1 and 52 for arm 2, respectively).

## Data Availability

The data presented in this study are available on request from the corresponding author, in compliance with current rules for the respect of privacy. The data are not publicly available due to privacy reasons.
